# The Influence of the Microbiome on Early-Life Severe Viral Lower Respiratory Infections and Asthma—Food for Thought?

**DOI:** 10.3389/fimmu.2017.00156

**Published:** 2017-02-16

**Authors:** Jason P. Lynch, Md. Al Amin Sikder, Bodie F. Curren, Rhiannon B. Werder, Jennifer Simpson, Páraic Ó Cuív, Paul G. Dennis, Mark L. Everard, Simon Phipps

**Affiliations:** ^1^Laboratory of Respiratory Mucosal Immunity, School of Biomedical Sciences, The University of Queensland, St. Lucia, QLD, Australia; ^2^Translational Research Institute, The University of Queensland Diamantina Institute, The University of Queensland, St. Lucia, QLD, Australia; ^3^The School of Agriculture and Food Sciences, The University of Queensland, St. Lucia, QLD, Australia; ^4^School of Paediatrics and Child Health, University of Western Australia, Perth, WA, Australia; ^5^Australian Infectious Diseases Research Centre, The University of Queensland, St. Lucia, QLD, Australia

**Keywords:** viral lower respiratory tract infection, RSV, asthma, microbiome, microbiota, microbiota and immunity, PVM

## Abstract

Severe viral lower respiratory infections are a major cause of infant morbidity. In developing countries, respiratory syncytial virus (RSV)-bronchiolitis induces significant mortality, whereas in developed nations the disease represents a major risk factor for subsequent asthma. Susceptibility to severe RSV-bronchiolitis is governed by gene–environmental interactions that affect the host response to RSV infection. Emerging evidence suggests that the excessive inflammatory response and ensuing immunopathology, typically as a consequence of insufficient immunoregulation, leads to long-term changes in immune cells and structural cells that render the host susceptible to subsequent environmental incursions. Thus, the initial host response to RSV may represent a tipping point in the balance between long-term respiratory health or chronic disease (e.g., asthma). The composition and diversity of the microbiota, which in humans stabilizes in the first year of life, critically affects the development and function of the immune system. Hence, perturbations to the maternal and/or infant microbiota are likely to have a profound impact on the host response to RSV and susceptibility to childhood asthma. Here, we review recent insights describing the effects of the microbiota on immune system homeostasis and respiratory disease and discuss the environmental factors that promote microbial dysbiosis in infancy. Ultimately, this knowledge will be harnessed for the prevention and treatment of severe viral bronchiolitis as a strategy to prevent the onset and development of asthma.

## Introduction

Viral lower respiratory infections (vLRI) caused by respiratory syncytial virus (RSV) are a major cause of morbidity and mortality in young infants. In developed countries, epidemiological studies have reproducibly implicated RSV-bronchiolitis as a major risk factor for the onset and progression of asthma [reviewed extensively in Ref. (1)]. The immunopathology associated with RSV-bronchiolitis in early-life may lead to long-lived alterations to airway structural and/or resident immune cells that render the host susceptible toward allergic sensitization and asthma (2–4). Susceptibility to both RSV-bronchiolitis and asthma has been attributed to defects in immunoregulatory and antiviral pathways, the cause of which may be genetic and/or environmental (1, 2, 5, 6). Interestingly, rates of RSV-bronchiolitis peak at 2–6 months of age (7, 8), whereas asthma typically begins to develop later in childhood (2), highlighting a window of susceptibility to RSV in early-life, which, if engaged, may leave the host vulnerable to asthma development. Hence, factors that influence the development and/or function of the immune system may critically impact the host response to RSV. It is now recognized that the composition and diversity of the microbiota, which stabilizes around 12 months of age in humans (9–13), fundamentally affects host physiology and immunity (12, 14–19). Several recent studies have demonstrated changes in the respiratory microbiome in subjects with RSV-bronchiolitis (20–23). Similar observations have been reported in asthma (24–28). Whether microbial dysbiosis—changes in the composition of the gut and/or lung microbiota—predisposes to RSV-bronchiolitis and asthma or is simply a consequence of disease remains unclear. In this review, we discuss recent clinical and experimental findings implicating a role for the microbiota in infant respiratory health and disease. We also consider how this knowledge might be harnessed for the prevention and treatment of severe bronchiolitis as a strategy to curtail the short- and long-term burden of disease, with a specific emphasis on the onset and development of asthma.

## The Link Between Severe or Frequent vLRI and Subsequent Asthma Development

Numerous birth cohort studies have assessed the association between vLRIs in early-life and asthma risk (29–46). Although this association is moderate for certain viruses [e.g., influenza (47) and human metapneumovirus (44, 48–50)], the association is much stronger for RSV and rhinovirus (RV) (29–42). This relationship also appears to extend to allergic sensitization, but whereas severe RSV vLRIs appear to precede allergic sensitization this is less common for RV; rather allergic sensitization is a precursor to severe RV vLRIs (51, 52). A recent study evaluating two large cohorts of infants (>80,000 and >180,000) found that the population-attributable risk for asthma contributed by infant bronchiolitis during the RSV season was 10 and 13%. However, in the subset of children with infant RSV-bronchiolitis, the attributable risk was 49 and 47% (41). The relationship between RSV disease severity and subsequent asthma inception is dose-dependent lending support to a causal relationship (42, 52, 53). In further support of this notion, RSV-immunoprophylaxis is effective in reducing rates of recurrent wheeze and asthma (54–56). However, because all humans have been infected with RSV by the age of 3 and the population at large is not asthmatic, other co-factors (e.g., genetic and/or environmental) likely collude with RSV, to increase the risk of bronchiolitis in infancy. Thus the asthma-prone phenotype is likely established early in life and progresses to persistent disease following subsequent rounds of allergen and/or viral exposure (2). Using a high-fidelity mouse model, we have demonstrated that defects in plasmacytoid dendritic cells (pDCs) or toll-like receptor (TLR) 7/interferon regulatory factor (IRF) 7 signaling predisposes to severe viral bronchiolitis and subsequent asthma (57–61). In this scenario, the impaired immune response may lead to enhanced epithelial injury and secondary tissue repair leading to long-lived changes to the epithelium (genetic or epigenetic). In turn, this may promote the development of Th2 immunity by modulating the underlying network of DCs and innate lymphoid cells (ILCs). We and others have shown that house dust mite (HDM)-induced epithelial-derived IL-1α and high-mobility group box (HMGB) 1 act in a cytokine cascade and in feed-forward loops to induce the production of Th2-instructive cytokines, such as IL-33, thymic stromal lymphopoietin, IL-25, and granulocyte-macrophage colony-stimulating factor (GM-CSF), which license local DCs and type 2 ILC to promote Th2 immunity (62–64). Hence, epithelial stress as a consequence of allergen exposure or respiratory viral infection-associated cytopathology in predisposed individuals leads to the release of tissue alarmins—IL-1α, HMGB1, and IL-33—to promote a cytokine microenvironment that is conducive to the generation of Th2 immunity. In addition to providing pro-type 2 inflammatory signals, these cytokines also act by inhibiting pro-type I inflammatory signals. For example, allergen-induced IL-33 amplifies the allergic response in part by inhibiting CD11b^+^ DCs (DC2) IL-12p35 production (65). IL-33 can also suppress the production of interferon alpha, suppressing its negative regulatory effects on type 2 immunity (59, 66).

The teleological role of Th2 immunity is to initiate tissue repair; however, following repeated environmental insult(s) throughout life, type 2 inflammation may lead to pathologic tissue remodeling (67–69). Once established, this inflammatory response is maintained by both structural cells [e.g., airway epithelial cells (AECs), endothelial cells, and airway smooth muscle cells] and immune cells (e.g., type 2 ILCs, DCs, macrophages, and T helper cell subsets), which act in concert to produce an array of pro-inflammatory lipids, cytokines, and chemokines [recently reviewed in Ref. (4)]. The relative importance of type 2 ILCs and CD4^+^ Th2 in this process has been the subject of much interest and debate in the field with recent studies implicating type 2 ILCs in antigen presentation (70) and potentiating memory CD4^+^ Th2 cells (*via* IL-13-dependent licensing of DCs) (64). Intriguingly, “innate” CD4^+^ Th2 cells have been proposed. This novel cell subset can be primed locally in the lung by Th2-instructive cytokines, together with an activator of the signal transducer and activator of transcription 1 family of transcription factors, in the absence of antigen presentation (71).

Now, that a number of type 2 instructive cytokines have been identified, this has opened up opportunities to halt the progression and/or decrease the severity of asthma through the use of humanized monoclonal antibodies. Additionally, a better understanding of the factors that confer susceptibility to bronchiolitis and its nexus with asthma onset, may yield new opportunities for targeted intervention. Critically, this raises the possibility of primary prevention. Emerging evidence discussed in this review highlights the supportive influence of the microbiome on the maturing immune system. Thus perturbations to the microbiome, occurring prenatally or postnatally, could adversely affect host defense against RSV, and this might lead to long-term alterations as a consequence of aberrant programming (genetic or epigenetic) of structural and/or immune cells. In this paradigm, further exposure of these susceptible individuals to environmental triggers of asthma (e.g., allergen and/or viral infection) in later-life may progress the asthma-prone phenotype to established and persistent disease.

## The Airway Bacterial Microbiota is Dysbiotic in Asthma, but Why?

For centuries, the lower airways have been considered a sterile environment, a dogma based primarily on culture-based studies in which any culturable microbes from bronchoalveolar lavage (BAL) samples were considered to be contamination or of little clinical significance (72). In fact, the lung harbors an abundant and diverse microbial community (a microbiota) that is highly dynamic and underpinned by the immigration and emigration of microbes with every breath of air (approximately 7,000 L/day) and the occasional subclinical aspiration of the oropharyngeal contents (73–75). Culture-independent techniques involving high-throughput sequencing of the 16S rRNA gene, a highly conserved locus of the bacterial genome, has led to a revolution in our understanding of the airway microbiome. Using this approach, investigators have begun to probe the human airways in health and disease, and pioneering studies have revealed that the microbial community inhabiting the lower airways of asthmatics is indeed quite different from that of healthy subjects (25, 26, 28). Whether the asthmatic airways are more highly populated (i.e., grater bacterial load) remains unclear with some studies finding increases in abundance (25) and others reporting no difference compared to control (26). Several studies report that the airway microbiota, sampled by BAL (26, 76) or nasal swab (28), of mild-to-moderate asthmatics is enriched in members of the Gram-negative bacterial phylum *Proteobacteria* including the potential pathogens *Haemophilis, Moraxella*, and *Neisseria* (26, 28, 76) and reduced in the commensal phylum *Bacteroidetes* (28, 76). However, this profile appears to differ somewhat according to the inflammatory phenotype and/or severity status, as well as corticosteroid treatment (27). For example, in a study directly comparing the sputum microbiota in severe and non-severe asthma, Zhang and colleagues found that mild-to-moderate and, to a lesser extent, severe disease was associated with increased *Proteobacteria*, while both severe and non-severe asthmatic individuals had a lower abundance of *Bacteroidetes* (particularly *Prevotella* spp.) and *Fusobacteria* (76). Moreover, a greater abundance of *Streptococcus* spp., which are affiliated with the phylum *Firmicutes*, was uniquely associated with severe asthma. In this study, severe asthma was associated with blood and sputum eosinophilia, but not neutrophilia. Green et al. found that sputum neutrophilia and IL-8 levels directly correlated with the abundance of *Streptococcus* spp, as well as *Haemophilus* and *Moraxella* in severe asthmatics (77), while Goleva and colleagues reported that *Haemophilus parainfluenzae* was uniquely present in the airways of corticosteroid-resistant asthmatics (26). Additionally, a study comparing the sputum microbiome in chronic and persistent/severe asthmatics found reduced bacterial diversity combined with a high prevalence of *Haemophilus influenzae* in asthmatics with neutrophilic inflammation, whereas asthmatics with eosinophilic inflammation had abundant *Tropheryma whipplei* (78). One interpretation of these data is that disease severity and possibly the inflammatory profile or asthma phenotype relate to the composition or dysbiosis of the airway microbiota. Further work is needed to determine whether the presence or absence of certain microbial communities underpins different asthma phenotypes or whether these changes are secondary to the pathological environment characteristic of the asthma subtype. Whereas several studies have examined the lung microbiota in stable asthma, very few have examined the microbiota during acute exacerbation(s) of asthma. This is surprising, since 80% of asthma exacerbations are attributed to a viral infection (34, 79), predominantly a species of RV, which likely affects or is affected by the bacterial community of the airways. An interesting study in which healthy adult subjects were experimentally infected with RV revealed increases in the relative abundances of upper airway *H. parainfluenzae* and *Neisseria subflava* following infection (80), two potentially pathogenic bacterial species reported to be present in the lower airways in asthma. Whether the upper airway microbiota reflects the lower airways remains an unresolved question, but these findings do highlight a shift toward an asthma-like microbiota caused by RV infection, which may impact the mechanism by which RV causes asthma exacerbations. In an attempt to address this issue, Kloepfer et al. studied a population of 308 school-age children, half with asthma and half healthy controls using PCR-based analysis of nasal secretions for a period of 5 weeks during autumn over 2 consecutive years (81). The authors found that the presence and abundance of either *Moraxella catarrhalis* or *Streptococcus pneumoniae* at the time of RV infection was positively associated with the severity of RV-associated exacerbations. The authors speculated that the viral infection/exacerbation could lead to a lung environment that is conducive to colonization with potential pathogens.

In summary, it is evident that lung microbial dysbiosis represents a component of the asthmatic syndrome; however, the nature of this dysbiosis and its relationship to asthma phenotype(s) remain ill-defined and needs further investigation. More importantly, it remains an open question as to whether microbial dysbiosis precedes and is causal for asthma onset or simply develops as a consequence of the dysregulated lung environment. In this regard, we must consider the early-life origins of asthma.

## Airway Microbial Dysbiosis and the RSV-Bronchiolitis to Asthma Nexus

The notion that asthma risk may be influenced by inappropriate colonization by microbes stems from the seminal findings of the Copenhagen Birth Cohort Study (82). Using culture-dependent profiling, this study was the first to uncover the presence of potentially pathogenic species in the oropharynx of 1-month-old infants significantly at risk of developing asthma in later childhood. Pathogenic species detected in these subjects included *M. catarrhalis, H. influenzae*, or *S. pneumoniae*. Additional studies by this group that showed these same bacterial species were present during periods of episodic wheeze caused by infection with a respiratory virus (83). These paradigm-shifting studies implicated colonization of the airways by opportunistic pathogens in the inception of asthma. Whether these bacterial species are causative for later asthma is not yet clear and remains a hotly debated subject (84–86). Some investigators argue that if pathogenic bacteria were causative then antibiotic therapies should reduce wheezy episodes and decrease asthma risk, but meta-analyses of population cohort studies suggest they do not (85–87). However, this interpretation is oversimplified since antibiotic treatment also ablates bacteria that are beneficial, for example those species which generate metabolites that promote the differentiation of regulatory T (Treg) cells (discussed later in more detail). Modern 16S RNA gene sequencing has revolutionized this area of research and continues to provide new insights. Recent studies armed with this technology have begun analyzing airway and gastrointestinal samples from birth cohort studies (22, 23, 88). Such analyses have also attempted to address important questions surrounding which bacterial species first colonize the airways and whether this differs in settings of acute respiratory illness and asthma risk. For example, an elegant study by Teo et al. sought to address this gap in knowledge in a recent study of children enrolled in the Childhood Asthma Study (22). In this study, nasopharangeal aspirates were collected from subjects during planned visits at 2, 6, and 12 months of age and within 48 h from the onset of an acute respiratory infection. This high intensity sampling regimen allowed the authors to temporally assess changes in the nasopharangeal microbiota and the relationship between upper airway colonization and chronic wheeze outcome at age 5. They found that *Staphylococcus* and *Corynebacterium*, common components of the human skin microbiome, were the dominant bacterial species present in the first 2 months of life before switching to either *Alloiococcus* or *Moraxella* at 6–12 months, concomitant with a stabilization of the microbiome. Because of the presence of species typically found on the skin, the authors speculated that infants are likely to be colonized initially with skin-dwelling bacteria from their parents and others, and that these founder populations are replaced over time by *Moraxella* or *Alloiococcus*. This upper respiratory microbiota then remains stable over time in healthy individuals. However, in infants suffering from virus-associated acute respiratory infection(s), a greater abundance of potential pathogens *Streptococcus, Moraxella*, and *Haemophilus* was observed, verifying earlier findings (82). Interestingly, when examining upper and lower respiratory infections separately, the authors found that early *Moraxella* colonization was associated with younger age of first upper respiratory infection, whereas early *Streptococcus* colonization was associated with earlier initial lower respiratory infection. Additionally, the level of subsequent asthma risk in these infants appeared to relate inversely with the age at initial *Streptococcus* colonization, leading the authors to speculate that tissue damage mediated by *Streptococcus* and/or infection with RSV/RV during this early-life period might have long-term effects that predispose to later asthma. A recent study in mice found that infection of 1-week-old neonates with a non-lethal dose of *S. pneumoniae* (D39) followed by aluminum hydroxide (alum)/ovalbumin (OVA) administration and OVA challenge in adulthood led to Th17 type inflammation, neutrophilia, and airway hyperreactivity (AHR) (89). Type 2 inflammation was also observed but did not correlate with increased AHR or gross lung pathology in co-exposed mice. Rather, the exaggerated Th17 phenotype was associated with impaired accrual of lung FoxP3^+^ Treg cells which typically suppress pathogenic effector T cell driven inflammation, including that mediated by Th17 cells. The function of these Treg cells was not assessed in this study and the nature of this altered immune response and tissue damage remains unclear. Nevertheless, these data suggest that *S. pneumoniae* exposure in early-life may act to dampen Treg cell responses, rendering the host susceptible to allergic airway inflammation. Because reduced numbers of Tregs have been observed in infants with RSV-bronchiolitis (90), and animal models have revealed a crucial role for these cells in regulating the inflammatory response to RSV (91), one possibility is that early colonization of the respiratory tract with *S. pneumoniae* dampens Treg cell responses thereby enhancing the severity of RSV disease, and in turn predisposing to allergic sensitization and asthma.

Notably, the study by Teo et al. also found that the presence of *Moraxella* was associated with increased severity of RSV infection. The relationship between RSV and *Moraxella* co-infection is an interesting one. Both virus and bacterium are more abundant during cooler months, and co-infection has been associated with a greater incidence of otitis media (92), suggesting that RSV infection may alter the upper respiratory microbiome of the infant host in a way that is conducive to bacterial infection (e.g., *Moraxella*). A recent study by Mansbach and colleagues explored this idea in a prospective cohort of >1,000 infants at 3 months of age (23). Of these infants, the major viral pathogen detected was RSV with a smaller proportion of subjects exhibiting a co-infection with RV, and a smaller proportion still infected with RV alone. However, healthy controls were not included in this study. Comparing the nasopharyngeal microbiota of these three groups, the authors found a high level of Firmicutes and the genus *Streptococcus* and a low level of Proteobacteria and the genera *Haemophilus* and *Moraxella* in the RSV-only group, while the opposite was true for the RV-only group and the RSV/RV co-infection group exhibited intermediate abundances of these phyla and genera. This pattern of specific virus and bacteria detected in the upper airways is akin to that observed by Teo and colleagues. However, it is not yet sufficiently clear whether these pathogenic bacteria contribute to disease or are simply reflective of opportunistic colonization/expansion in individuals with more severe illness. This question requires further work using both clinical cohorts and translational animal models of disease. The latter will be fundamental to unraveling the complex interplay between host, virus and bacteria (airway microbiota and pathogens) and the long-term consequences for asthma risk.

## Microbial Colonization and the Development of Early-Life Respiratory Disease

Admission rates for bronchiolitis (93) and the prevalence of asthma and allergies have increased substantially over the past 30 years (94–96). This has implicated a key role for environmental factors, most notably those associated with a “Western lifestyle.” Initial evidence for this was garnered from epidemiological studies in Europe linking decreases in family size and increased personal hygiene with allergic disease development and led to the generation of the “hygiene hypothesis” (97, 98). The central tenet of the hygiene hypothesis is that components of the mammalian immune system fail to develop appropriately or become dysregulated as a consequence of insufficient exposure to microbes. Subsequent studies of European children growing up in rural environments have strongly supported and extended this concept. For example, Reidler et al. demonstrated in a cross-sectional study that children who grew up in rural farming environments were less likely to develop allergy and asthma and that disease risk was even lower if the mother had also experienced this microbe-rich environment during pregnancy (99). Other studies in farming communities have shown that high levels of lipopolysaccharide (LPS) in barn-dust and gastrointestinal exposure to unpasteurized milk are associated with tolerance to ubiquitous allergens and the prevention of atopy (100, 101). The protective “farm effect” on childhood asthma has been reproduced in several studies around the world, and a recent meta-analysis concluded that an overall 25% reduction in asthma risk can occur despite heterogeneity in the study populations (102). However, the strongest protection from childhood asthma was recently reported in Amish populations in the US, which retain traditional farming methods (e.g., use of horses for transportation, single-family dairy farms) (103). By contrast Hutterites living in the US, with otherwise similar genetic background and lifestyle factors, embrace modern farming technologies (e.g., highly industrialized, communal farms). Among these children, the prevalence of asthma in Amish compared to Hutterite school children was 5.2 versus 21.3% and the prevalence of allergic sensitization was 7.2 versus 33.3%. Interestingly, LPS levels were 6.8 times higher in Amish homes than in Hutterite homes, a finding that was associated with greater numbers of peripheral blood neutrophil which expressed lower levels of the activation markers (CD11b, CXCR4, and CD11c), lower numbers of peripheral blood eosinophils and lower production of the Th2-associated cytokines IL-33, IL-25, IL-5, and IL-4 by peripheral blood lymphocytes in the Amish children compared to the Hutterite children. By exposing mice to Amish or Hutterite house dust extracts prior to performing an i.p. OVA model of allergic airway inflammation, the authors showed that the high LPS levels of the Amish house dust conferred protection from AHR and airway eosinophilia, whereas the Hutterite house dust did not. As had been shown in a previous report of European farm children (104), the beneficial effect of house dust from the Amish children was associated with higher expression of the A20 gene (TNFAIP3) in peripheral blood lymphocytes; however, an increase in IRF7, a hub gene and master regulator of type-I IFN production, was also found in Amish children. Hence, it will be important to determine whether the beneficial effects of farm house dust exposure extend to protecting against vLRI and viral exacerbations of asthma.

Further evidence for the role of local microbial exposures protecting against allergic disease development in early-life comes from studies of microbial exposures associated with pet ownership. For example, one study showed that exposure to dogs and to a lesser extent cats in infancy is associated with protection against the development of allergies in childhood, although asthma rates were not examined (105). Notably, environments with no household pet exhibit low levels of bacteria in the local environment (105–107), and thus in contrast to farm environments, these “microbe low” residences confer a higher risk of allergic disease development. Intriguingly, these homes are reported to possess a wider range of fungal species (108), which have long been associated with the development of asthma (109), although whether they shape the microbiome in early-life in a way that renders the host susceptible toward the development of allergies and asthma in later childhood remains to be determined.

These findings have fueled experimental animal studies to establish the mechanisms underlying the link between microbial exposure and protection from asthma. For example, a recent study by Schuijs et al. examined the beneficial effects of barn-dust in an acute HDM model of allergic airway inflammation in mice (104). In this murine model, a key initial step in the process of sensitization to the HDM is allergen-induced activation of AECs through TLR4. TLR4-signaling leads to the secretion of the chemokine CCL20 and GM-CSF, both of which are required for the recruitment and functional maturation of airway DCs. Consistent with the results of the European epidemiologic barn-dust studies, Schuijs and colleagues found that airway exposure to LPS before or during HDM exposure markedly attenuated the asthma-like pattern of pro-inflammatory responses to HDM and reduced the activation of local DC functions mediated by CCL20 and GM-CSF. They further demonstrated that exposure to LPS suppresses AEC responsiveness to TLR4-induced activation by HDM and that this suppression is dependent on the attenuation of signaling by nuclear factor κB (NF-κB). This effect was mediated by an increase in the synthesis of the enzyme A20, encoded by the *Tnfaip3* gene, in AECs. *Ex vivo* cultures of human bronchial epithelial cells revealed a similar inverse association between LPS-mediated stimulation of GM-CSF production and activation of the TNFAIP3 gene. This association was further examined in a case–control study in which the investigators observed a relative deficiency in the LPS-mediated TNFAIP3 gene expression in AECs from asthmatic subjects as compared with healthy controls. Moreover, the authors observed a positive association between a polymorphism in the TNFAIP3 gene and susceptibility to asthma in the cohort of GABRIELA [Multidisciplinary Study to Identify the Genetic and Environmental Causes of Asthma in the European Community (GABRIEL) Advanced Study]. Thus, exposure to aerosolised bacterial components appears to regulate the threshold for AEC activation, highlighting A20 expression by AECs as a potential therapeutic target for asthma. Although, the effects of A20 knockdown in AECs abrogated LPS-mediated protection against the airway pathology induced by HDM, CCL20, and GM-CSF were not fully suppressed. This suggests the existence of other pathways by which LPS suppresses asthma. This apparent heterogeneity is not unexpected and perhaps reflects the complex nature of the causal pathways underlying asthma development, particularly in relation to other environmental stimuli.

## Microbiota Hypothesis

Following birth, the infant is exposed to a diverse host and environmentally associated microbiota. During natural birth, the infant is principally colonized by a founder community of microbes derived from the mother’s vagina and feces (13, 110). Postnatally, breast milk further diversifies the neonatal microbiota by providing secretory immunoglobulin (Ig)A, prebiotic glycans (e.g., milk oligosaccharides), and other microbial metabolites [e.g., short-chain fatty acids (SCFAs)] (12, 111, 112). Cesarean birth and formula feeding disturb the natural process of colonization. In offspring born by Cesarean section, the predominant bacterial populations in gut closely align with that of the mother’s skin (111, 113), an effect that it is further compounded in formula-fed infants (114). The observation that formula-fed infants are at greater risk of chronic inflammatory and metabolic diseases indicates that prebiotic- or probiotic-supplemented infant formulas do not fully replicate the effect of breast milk on the development of the neonatal microbiota (115). This highlights that our knowledge of the factors that influence the emergence of the neonatal microbiota remains incomplete. Nevertheless, it is now appreciated that the composition of the intestinal microbiota is highly plastic during the early-postnatal period and into early childhood, changing rapidly in response to environmental interventions, including invasion by pathogenic microorganisms, antibiotic treatment, and diet (116), all of which likely affect immune homeostasis. Thus, the hygiene hypothesis has now been extended to incorporate a wider array of environmental conditions that can perturb the microbiome, especially in the gastrointestinal tract. This conceptual evolution has led to a new theory coined the “microbiota hypothesis.” Studies of gnotobiotic mice [also called germ-free (GF)] mice have established a key role for the host microbiota in influencing the development and function of the immune system GF mice exhibit several gross physiological and functional abnormalities in the gastrointestinal tract including enlarged cecum, reduced gastrointestinal motility, and reduced production of antimicrobial peptides (117, 118). The absence of commensals also has profound effects on the development of the immune system, including defects in lymphoid tissue development within the spleen, thymus, and peripheral lymph nodes (119). Of relevance, an elegant study by Olszak and colleagues found that invariant natural killer T cells accumulate in the colonic lamina propria and lung in GF mice, resulting in increased morbidity in models of inflammatory bowel disease and allergic asthma as compared with that of specific pathogen-free mice (120). Notably, the elevated expression of the chemokine ligand CXCL16 in the lungs of GF mice could be normalized by conventionalization (introduction of GF mice to SPF conditions) but only during the first 2 weeks of life, an effect that was dependent on microbiota-induced epigenetic modifications at the *Cxcl16* locus. Conventionalization after this 2-week period failed to rescue the asthma phenotype, highlighting a critical window of susceptibility during the postnatal period. Beyond the *Cxcl16* gene, it will be important to elucidate the genetic or epigenetic mechanisms by which the microbiota programs airway immune and structural cells as this appears to fine-tune the host response and affect not just the initial encounter with an environmental stimuli, such as an allergen or pathogen, but also the development of long-term immunity.

It is emerging that microbial metabolites play a key role in regulating host physiology and immunity. Herein, we focus on the SCFAs acetic acid, butyric acid, and propanoic acid; however, it is noteworthy that other lipid derivatives are known to have immunomodulatory effects [reviewed in Ref. (121)]. SCFAs range in concentration from 50 to 100 mM in the gut and are the main metabolic end products from bacterial fermentation, particularly fermentation of dietary fiber. Hence, changes in dietary habits and factors that perturb the microbiota such as antibiotic use have a profound effect on SCFA production (122, 123). In the gastrointestinal tract, SCFAs represent an important energy source for both the local microbiota and intestinal epithelial cells. But, as well as being local substrates for energy production, it is now appreciated that SCFAs have diverse regulatory functions in host physiology and immunity affecting both hematopoietic and non-hematopoietic cells. SCFAs function as histone deacetylase (HDAC) inhibitors and ligands, predominantly agonists, of G protein-coupled receptors (GPRs). The ability of SCFAs to inhibit HDACs generally promotes a tolerogenic, anti-inflammatory cell phenotype necessary for the maintenance of immune homeostasis, and this functional property supports the concept that the gut microbiota can act as an epigenetic regulator of host physiology. Exposure of peripheral blood mononuclear cells or neutrophils to acetate, propionate, or butyrate suppresses NF-κB and downregulates the production of the pro-inflammatory cytokine TNF-α (124, 125). Subsequent studies revealed that these anti-inflammatory effects, mediated by HDAC inhibition, extend to both macrophages (126, 127) and DCs (128, 129). SCFAs can also modulate T cells, particularly Treg cells, through HDAC inhibition. For example, inhibition of HDAC9 increases FoxP3 expression, enhancing the suppressive function of Treg cells during homeostasis and in mouse models of colitis (130). Studies characterizing the ability of specific SCFAs to regulate the quality of the colonic Treg cell pool have shown that propionate and butyrate induce FoxP3 in an HDAC-dependent manner, while acetate is less effective (15, 16). Of direct relevance to the function of SCFAs in asthma, Thorburn and colleagues explored the effect of modifying dietary fiber intake on the susceptibility of mice toward HDM-induced allergic airway inflammation (131). They began by comparing the gut microbiota of mice fed a high-fiber diet to mice fed an equicaloric control diet or a no-fiber diet for 3 weeks. As expected, the high-fiber diet significantly increased circulating levels of acetate and propionate, a phenotype that was associated with greater representation of *Bacteroides, Clostridium*, and *Pandoraea* spp. compared to mice fed the control diet. Mice fed on a no-fiber diet exhibited reduced (although not statistically significant) circulating SCFA levels and a greater abundance of Proteobacteria compared to the control diet group. When the effect of diet on HDM-induced allergic airways inflammation was assessed, mice fed a high-fiber diet or treated with acetate in the drinking were protected exhibiting reduced levels of airway eosinophils, type 2 cytokine production (IL-4, IL-5, and IL-13), serum IgE, and AHR. The no-fiber diet did not alter susceptibility to asthma in this model, consistent with the lack of effect on SCFA levels, which was unexpected given previous findings from another group (129). Because asthma typically develops in childhood, the authors next explored the effect of their dietary perturbations or acetate exposure during late-stage pregnancy (from E13) until weaning (3 weeks of age). The offspring of mice fed a high-fiber diet were protected from HDM-induced allergic airway inflammation whereas the offspring from mice fed a no-fiber diet developed disease to the same degree as mice fed the control diet. This latter finding is quite surprising given the importance of the gut microbiota and circulating SCFAs for immune development in early-life. A possible explanation stems from the use of a high-dose of HDM which likely induced a maximal response in the control fiber diet fed mice, and hence this response could not be further elevated in the group fed on a no-fiber diet. Nevertheless, the effectiveness of the high-fiber maternal diet suggests that protective effects can occur *in utero*. In support of this, the investigators found that either acetate administration or high-fiber diet feeding until birth was sufficient to protect the offspring. This finding was further confirmed by litter swapping experiments, although the effect was less profound, for example, IL-5/13 production from lymph node T cell cultures was unchanged in the high-fiber group, suggesting that some of the protective effects of the high-fiber diet occur postnatally. To translate their findings, the authors next explored whether human SCFA levels correlated with fiber intake in pregnant mothers and wheeze in their offspring. A significant but weak inverse correlation was observed between circulating maternal acetate levels and the percentage of infants requiring two or more general practitioner visits for wheeze and cough. Although viruses are a major cause of wheeze in infancy, the authors did not report which respiratory viruses were detected in these subjects. Importantly, it remains unknown whether lower SCFA levels, because of changes in maternal diet or other postnatal perturbations, increase the risk of respiratory viral illness, a major risk factor for asthma. Since the high-fiber diet conferred protection *in utero* and this persisted into adulthood the investigators hypothesized that this effect might be due to epigenetic regulation of Treg cells. They found that, through HDAC9 inhibition, acetate treatment endowed greater numbers of Treg cells and increased the suppressive capacity of these cells. However, it remains to be tested whether a high-fiber diet also mediates this effect specifically *via* acetate. The authors speculated that propionate played a less important role in their model, primarily because it was found at lower concentrations in the circulation; however, propionate is also a potent HDAC inhibitor in Treg cells, and other investigators have demonstrated a key role for propionate in protecting against HDM-induced asthma (129). Thus, the *in utero* effects of propionate remain ill-defined.

## Short-Chain Fatty Acids and Asthma

**Figure 1:**
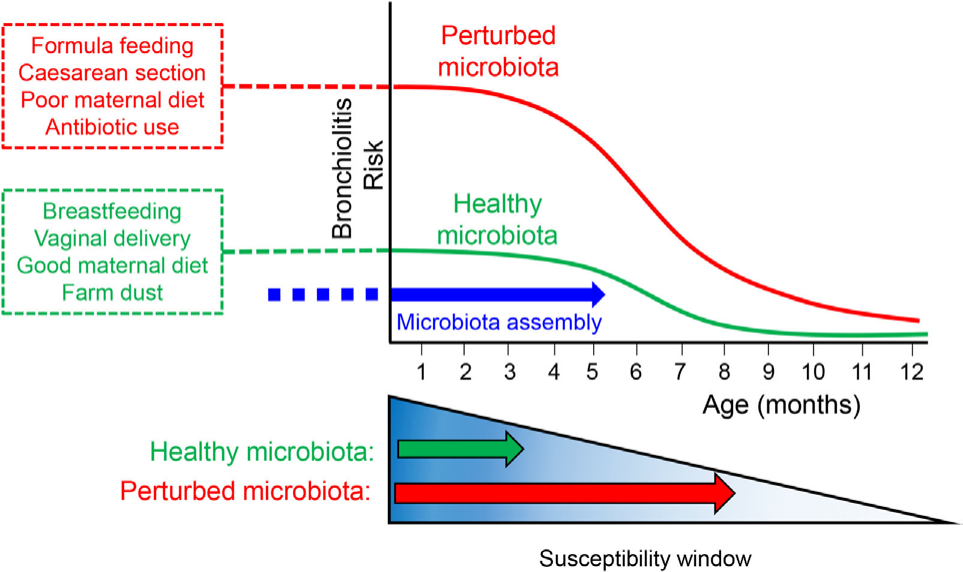
**Schematic showing the timing of microbiota assembly with respect to bronchiolitis risk within the first year of life and how perturbations to the maternal or neonatal microbiota might prolong the window of susceptibility to bronchiolitis and increase overall risk**.

As well as their ability to inhibit HDAC activity, several studies have determined that SCFAs affect host immunity *via* engagement of GPRs, including OLFR78, GPR43 (FFAR2), GPR41 (FFAR3), and GPR109A (HCAR2). These receptors are expressed by numerous cell types, including immune cells and intestinal epithelial cells and are activated by various endogenous agonists including those summarized in Table 1. GPR43 signaling plays an important role in SCFA-induced neutrophil chemotaxis (124) and the expansion and suppressive function of Treg cells induced by SCFAs. GPR41 has aslo been implicated in supporting host immune function (132). In wild-type mice, but not in GPR41-deficient mice, SCFAs arrest the maturation of pro-inflammatory DCs in a GPR41-dependent mechanism, thereby preventing the development of allergic airway inflammation in mice (129). It is noteworthy that a recent study reported mRNA and protein expression of GPR41 and GPR43 in bronchial epithelial cells (133), raising the possibility that SCFAs may regulate the airway epithelium, although functional studies remain to be performed. Studies in man have begun to test the hypothesis that decreases in gut microbial SCFAs antedate asthma development. In a recent high-profile study of Canadian infants, acetate levels in feces and serum were found to be lower in atopic/wheezy infants (at high risk of childhood asthma) compared to healthy controls at 3 months (but not 12 months) of age (134). This was associated with reductions in specific bacterial genera: *Faecalibacterium, Lachnospira, Veillonella*, and *Rothia* in the feces. Similarly, in a cohort of Swedish infants, the composition and diversity of the gut microbiota during the first month of life was significantly lower and associated with asthma diagnosis at 7 years of age (135). Collectively, these data suggest that a lack of gut microbial diversity in early-life has long-term consequences on host immunity. However, the direct cause of low infant SCFA levels in these studies remains unclear and may stem from a combination of factors affecting the maternal and neonatal microbiota. It will be important to address whether low SCFA levels are causally linked to vLRIs and asthma development and therein the underlying mechanisms. Additionally, whether poor diet and perturbations to the gut microbiota increase bronchiolitis risk as a consequence of a reduction in circulating SCFA levels remains to be determined; however, one might speculate this would have important long-term consequences for predisposition toward asthma and possibly other chronic respiratory diseases such as chronic obstructive pulmonary disease. In this regard, establishing whether SCFAs enhance lung epithelial barrier integrity is likely to be of great importance. Given the accumulating data discussed in this review linking vLRIs and microbiota-associated perturbations in early-life it will be important to elucidate the biological mechanisms underlying these clinical observations. We propose that maternal and/or infant microbial dysbiosis increases and prolongs the window of bronchiolitis risk in infancy (see Figure 1); this may occur through functional aberrancy of the host response to initial RSV infection, leading to severe illness, and this alone may be sufficient to cause long-lasting changes to host immunity that predisposes to asthma in later life. In this proposed scenario, environmental factors that affect microbial colonization in the infant, such as maternal diet, cesarean delivery, transmission of microbial dysbiosis from the mother, maternal/infant antibiotic use, breast-feeding, as well as others will impact on the production of microbial metabolites and as a consequence affect the development and function of immune cells (e.g., DCs and Tregs) and structural cells (e.g., AECs) that in turn, control RSV immunopathogenicity.

**Table 1:** **Principal receptors for short-chain fatty acids**.

Receptor	Selective agonist (pEC_50_)	Reference
OLFR78	Propionic acid (3.0)	Pluznick et al. (136)
GPR43 (FFAR2)	Propanoic acid (3.0–4.9)	Brown et al. (137), Le Poul et al. (138), Nilsson et al. (139), and Schmidt et al. (140)
	Acetic acid (3.1–4.6)	
	Butyric acid (2.9–4.6)	
	1-Methylcyclopropanecarboxylic acid (2.6)	
	Trans-2-methylcrotonic acid (3.8)	
GPR41 (FFAR3)	Propanoic acid (3.9–5.7)	Brown et al. (137), Le Poul et al. (138), Nilsson et al. (139), Schmidt et al. (140), and Xiong et al. (141)
	Butyric acid (3.8–4.9)	
	1-Methylcyclopropanecarboxylic acid (3.9)	
	Acetic acid (2.8–3.9)	
GPR109A	β-d-Hydroxybutyric acid (3.1)	Taggart et al. (142)

## Breast-Feeding and Asthma

As well as being the primary source of nutrition for the infant, breast milk contains immunomodulatory factors that affect the host immune response. The intestinal contents of breast-fed infants are acidic (pH 5.0), whereas those of non-breast fed infants is neutral (pH 7.1) (143). Milk oligosaccharides are the principal factor that support the growth of bifidobacteria (112) and hence may influence gastrointestinal and circulating SCFA levels in the infant. Thus, milk oligosaccharides likely serve as prebiotics to support the selective growth of “beneficial” bacteria, providing these commensals with a competitive advantage over potential pathogens. Milk oligosaccharides have also been postulated to exert direct anti-pathogenic and pro-tolerogenic roles in the intestine by acting as glycan receptor decoys for microbial adhesion factors and preventing pathogen attachment. Additionally, they can modulate the balance between Th1/Th2 immunity and provide essential nutrients for brain development and cognition [for a detailed review, see Ref. (112)]. Breast milk also contains a variety of food borne and aeroallergens that are transferred to the infant and influence sensitization to these antigens. In an elegant study by Verhasselt and colleagues, transforming growth factor-β transferred in breast milk was shown to facilitate T cell tolerance, protecting the offspring from OVA/alum-induced allergic airways disease (144). Birth cohort studies demonstrate that non-breast-fed infants are at greater risk of developing asthma (145–148), strongly implicating maternal factors in protecting against disease. Moreover, several epidemiological studies have linked a lack of breast-feeding to the severity of RSV infection (149–152), although this finding is not universal (153). Taken together, these studies demonstrate that breast milk represents a critical environmental determinant of colonization and profoundly influences immune homeostasis and postnatal development. However, the specific breast-milk derivatives and the causal pathways relating these to protection against respiratory pathogens and allergic sensitization in early-life require further investigation.

## The Maternal Microbiota and Infant Respiratory Health

The composition of the maternal intestinal microbiota and the infant intestinal microbiota has been shown to affect the risk of infant wheeze (88), although the underling mechanisms have remained elusive. In an elegant study designed to address whether transient colonization during gestation alone can affect immune cell development and immune responses in the offspring, de Agüero and colleagues inoculated pregnant GF mice during late gestation with a bioengineered strain of *Escherichia coli* (HA107) that is cleared from the intestine within 72 h (154). Interestingly, colonization with *E. coli* during late-gestation led to a more competent intestinal immune system in the offspring, including increases in type-3 ILCs and F4/80^+^ CD11c^+^ mononuclear cells. This more physiologically normal cellular phenotype attenuated the overt inflammatory response observed in GF mice in response to microbial molecules and penetration of intestinal pathogens. Importantly, the protective effect of gestational colonization was not restricted to the gastrointestinal tract as the investigators demonstrated improved orchestration of the immune response within the spleen following intraperitoneal LPS administration. These intriguing data, albeit from an experimental system, suggest for the first time that the systemic immune responses of the offspring can be shaped by maternal gestational colonization alone. Whether similar processes operate to control the seeding of ILC subsets at other mucosal surfaces remain to be determined. The bidirectional signaling between type 2 ILCs and epithelial cells in the respiratory tract is important for tissue homeostasis and repair (155), and so it will be important to learn whether the maternal microbiota can influence lung barrier immunity to respiratory pathogens and other stimuli. Mechanistically, de Agüero et al. found that gestation-only colonization conferred protection in part through the generation of maternal antibodies. However, maternal antibody transfer alone was not entirely sufficient to mediate the positive effects of gestational *E. coli* on immunity, rather it assisted in the transfer of bacterial metabolites including aryl hydrocarbon receptor ligands, known to drive ILC3 expansion (156) and limit adult bacterial translocation across the intestinal epithelium. Consistent with the literature, the authors found that aryl hydrocarbon receptor ligand administration restored the levels of ILC3 in the gut, although other peripheral tissues were not assessed. The authors found no role for SCFAs, as exogenous administration of a butyrate, propionate and acetate mixture during pregnancy had no effect on the numbers of type-3 ILCs and mononuclear cells in the offspring’s intestine, although the *in utero* effects of SCFAs on other aspects of immune responsiveness in the offspring was not assessed.

The influence of the maternal microbiota and provision of various constituents in the breast milk must now implicate an important role for the maternal diet in the early development and training of the offspring’s immune system. Of relevance, a recent prospective birth cohort study of >56,000 Argentinian children under 2 years of age examined maternal dietary preferences and the impact on the children’s respiratory health (7). Of the 1,293 children who had a respiratory infection, >60% were infected with RSV, 22% with human RV, and 4% with influenza, highlighting RSV as the most significant pathogen affecting infant respiratory health. Intriguingly, when the maternal diet was high in carbohydrates and low in fruit and vegetables, this had profound effects on RSV-associated disease in the offspring, increasing the odds ratio of developing severe hypoxemia and life-threatening disease. The mechanisms underpinning this association remain unclear but might relate to alterations in the maternal microbiome and possibly fetal development. Most notably, it will be important to ascertain the relative contribution of elevated carbohydrate intake versus low fiber intake on disease outcome, i.e., was severe RSV-bronchiolitis caused by a lack of fiber and the resulting decrease immune cell-supportive microbial metabolites (e.g., SCFAs) or refined sugars promoting the outgrowth of bacterial pathogens or a combination of both.

Understanding the processes by which maternal diet influences asthma susceptibility is of crucial importance. As discussed above, feeding pregnant mice a high-fiber diet or SCFA-supplementation *via* the drinking water suppresses the development of allergic airway inflammation by increasing the suppressive activity of Treg cells *via* a HDAC9-dependent mechanism (131). Importantly, the suppressive effects of the maternal high-fiber diet were passed on to the offspring, indicating the epigenetic potential of SCFAs in the development of the immune system and in protection from asthma. In addition, feeding adult mice with a high-fiber diet or treating with exogenous propionate is protective against HDM-induced airway inflammation (129). Collectively, these data highlight the potential for modulating SCFA levels as a preventative strategy to protect against RSV-bronchiolitis and asthma.

## Harnessing the Microbiota to Prevent and/or Treat Bronchiolitis

Immunological tolerance is an important goal for the prevention of asthma and allergic diseases and thus mechanisms to promote a healthy intestinal microbiota are under investigation (157–159). A new wave of Th2-targeted biologics has recently been approved for asthma (160); however, we speculate that this approach is unlikely to reverse the aberrantly programmed epithelium and/or immune response (61, 161). Microbial metabolites and beneficial commensals shape the immune response in early-life, suggesting that supplementing or mimicking these factors may represent a new approach for disease prevention. Of greater interest will be to determine whether such strategies can alter the course of disease or even reverse established asthma, a far greater challenge should the epigenetic programming of immune and structural cells already be set to an aberrant phenotype.

### Prebiotics, Probiotics, and Synbiotics

In the 2013 update to the Cochrane Review, a meta-analysis was performed on two studies examining the effect of prebiotic treatment on asthma outcomes (162, 163). In the first study, Moro et al. reported a significant reduction in asthma outcome following treatment of 102 infants “at risk” of allergies with galacto-oligosaccharides and fructo-oligosaccharides (0.8 g/dL) compared to 104 treated with placebo. In the second report, Westerbeek et al. treated similarly high-risk infants with acidic and neutral oligosaccharides (20/80%; 1.2 g/L) and found no significant difference in asthma outcomes. The meta-analysis reported no significant effect of the prebiotic treatment overall, although significant heterogeneity was found between these reports. One possible explanation for the discrepant findings is the prebiotic employed and their respective effects on the infant microbiota. Although both of these oligosaccharides have been shown to increase bifidobacterial growth, their impact on other potential beneficial gut microbes remains to be fully determined. Moreover, in both studies prebiotic treatment began at approximately 6 months of age though microbial dysbiosis has been reported at 3 months of age in infants that go on to develop asthma in later childhood (134) and may commence even earlier, as described above. Thus, an earlier intervention that positively affects the assembly of the host microbiota is likely to be more successful. The use of prebiotics as therapeutics and preventatives is in its infancy; however, the nature and timing of these prebiotic strategies will improve as our knowledge of the composition of the human microbiota and its functional effects on host physiology and immune development increases.

A meta-analysis of four studies investigating effect of probiotics on established asthma found little or no benefit (164). However, the authors highlighted that marked heterogeneity among the studies made direct statistical comparisons difficult. This meta-analysis did not include a subsequently published study by Chen et al. (165). In this double-blinded, placebo-controlled trial, oral administration of *Lactobacillus gasseri* over a 5-week period was found to be effective in reducing bronchial hyperreactivity, improving lung function, and reducing day-time asthma symptoms in school-age (6–12 years) children with allergic asthma (165). *L. gasseri* treatment decreased the production of pro-inflammatory cytokines (e.g., TNF-α, IL-13) from phytohemagglutinin- or Derp1-stimulated peripheral blood mononuclear cells, although unfortunately Treg cells were not measured as part of the study. Similarly, oral inoculation with *Lactobacillus rhamnosus* or *Lactobacillus reuteri* protected against allergic airway inflammation in mice by increasing the number and function of Treg cells in the systemic circulation (158, 166, 167). In another study, oral administration of the bacterial preparation OM-85BV ameliorated the hallmark features of allergic asthma in mice (IgE, airway eosinophilia, and AHR) *via* the induction of gastrointestinal Treg cells. These suppressive Treg cells trafficked to the lung and inhibited airway mucosal CD86^+^ DCs, blunting the type 2 inflammatory response (168). In the context of allergic disease, few studies have evaluated the clinical efficacy of a probiotic in combination with prebiotic oligosaccharides (known as a synbiotic). One randomized trial examined 29 adults with asthma and HDM allergy treated with synbiotics (*Bifidobacterium breve* M-16V and a mixture of 90% short-chain galacto-oligosaccharides and 10% long-chain fructo-oligosaccharides) or placebo for 4 weeks followed by a bronchial challenge with HDM allergen (169). Although peak expiratory flow and systemic Th2 cytokines were reduced in the synbiotic-treated group, there was no effect on sputum eosinophilia or neutrophilia. These preliminary findings require replication by other investigators.

Interestingly, pre-treatment of mice with *L. rhamnosus* has also been shown to protect against pathogenic type 2 inflammation during RSV infection. This was associated with an increase in the production of IFN-β, IFN-γ, and IL-10 as well as both CD103^+^ and CD11b high DCs, although Treg cells were not assessed (170). A number of clinical studies have demonstrated the efficacy of prebiotic (171, 172) or probiotic interventions (172–175) for the treatment of respiratory viral infections. For example, in a randomized, placebo-controlled, double-blind trial, Luoto et al. investigated the efficacy of galacto-oligosaccharide and polydextrose mixture (1:1), known to promote bifidobacteria and lactobacilli, and the probiotic *L. rhamnosus* in a cohort of preterm infants in Finland. Strikingly, both the prebiotic mixture of galacto-oligosaccharide and polydextrose and *L. rhamnosus* significantly reduced the incidence of RV-induced episodes, which was the cause of the infection in 80% of the infants in the cohort. Although RSV-associated vLRIs were not affected by either treatment, the RSV group represented only a small proportion of the cohort, and therefore, larger studies will be required to determine the effect of synbiotics on RSV-bronchiolitis risk. Moreover, it will be important for longer term studies to assess whether decreasing the severity of vLRI in these children can protect from the development of asthma in later-life.

### SCFAs and SCFA Mimetics

Human epidemiology and experimental studies in preclinical models of asthma suggest that dietary fiber protects against asthma risk by increasing fecal and systemic levels of SCFA. Accordingly, mimicking the anti-inflammatory effects of SCFAs pharmacologically is an attractive approach therapeutically. The anti-inflammatory effects of acetate and propionate have been ascribed to HDAC inhibition and the activation of GPR41 and/or GPR43 signaling, and as described above, these pathways ameliorate allergic airway inflammation in mice by increasing the function of Treg cells or by dampening pro-inflammatory DCs (15, 129, 131, 176). Although GPR109a, the primary receptor for butyrate, is reported to be expressed in lung tissue, its cellular distribution and function in the lung remains unclear (177). An additional target of SCFAs may be the bronchial epithelium, which expresses both GPR41 and GPR43, although increases in SCFA levels and GRP41 expression are proposed to be deleterious in the context of cystic fibrosis (133). Notably, recent pharmacological studies have reported development of small molecules with greater selectivity than endogenous SCFAs (140, 178), although increasing the potency of these compounds has yet to be achieved. Moreover, the efficacy of this current generation of molecules in animal models of asthma remains unexplored. Therefore, in addition to further elucidating the biology of these receptors, it will be important for drug discovery programs to continue to improve the selectivity and potency of synthetic agonists and to test their efficacy in clinically relevant models of viral bronchiolitis and asthma. pDCs present an attractive target for the prevention of severe RSV-bronchiolitis and asthma due to their capacity to produce vast amounts of type I IFN *via* engagement of TLRs (e.g., TLR7) and promote Treg cell expansion (179). We have shown that infection with pneumonia virus of mice (58), a mouse-specific Pneumovirus of the same genus as RSV, predisposes to severe bronchiolitis in *Tlr7*-deficient mice, while the adoptive transfer of *Tlr7*-sufficient pDC to *Tlr7*-deficient mice confers protection, implicating a critical role for TLR7 on pDCs. Moreover, secondary infection of TLR7-deficient mice induced the cardinal features of asthma, including AHR, airway remodeling (e.g., smooth muscle hyperplasia), and airway inflammation (180). Collectively, these findings suggest that the nexus between severe bronchiolitis and subsequent asthma is underpinned by perturbations to the pDC compartment (61). Hence, it will be important to determine whether SCFAs or SCFA mimetics are able to modulate pDC function.

## Conclusion

Illuminating epidemiological, clinical, and preclinical studies suggest that the microbiome plays a critical role in protecting against asthma and allergies, particularly in early-life. Despite the fact that RSV-bronchiolitis predominantly affects infants under 6 months of age, the influence of gut microbial colonization and microbial metabolites on susceptibility to severe RSV-bronchiolitis has yet to be investigated. Programming of airway immune and structural cells likely occurs during this window of susceptibility and will have long-term consequences for susceptibility toward asthma-inducing stimuli. Identifying the molecular pathways that link the gut microbiota and the development of lung mucosal immunity has the potential to identify new therapeutic targets for the treatment of RSV-bronchiolitis. In view of the strong link between severe RSV-bronchiolitis and subsequent asthma, such a strategy has the potential to ameliorate the current high rates of allergic asthma in developed nations.

## Author Contributions

JL performed background research and wrote the review. MS, BC, RW, and JS helped research the ancillary literature. PC, PD, and ME provided insightful discussion and edited the review. SP conceived the idea for the review and edited drafts.

## Conflict of Interest Statement

The authors declare that the research was conducted in the absence of any commercial or financial relationships that could be construed as a potential conflict of interest.
